# OD41 Conducting Systematic Reviews On Rare Diseases: Lessons Learned From The European Reference Networks Guidelines Programme

**DOI:** 10.1017/S0266462324001697

**Published:** 2025-01-07

**Authors:** Carmen Martín-Gómez, Rebeca Isabel-Gómez, Guillermo Pérez García, Maria José Vicente Edo, Aythami De Armas-Castellano, Mar Trujillo-Martín, Anna Godo Pla, Maria Dolors Estrada Sabadell, Josune Domínguez García, Ainhoa Jausoro

## Abstract

**Introduction:**

A consortium of five Spanish health technology assessment (HTA) agencies conducted the European Reference Networks Guidelines Programme for the development, appraisal, and implementation of clinical practice guidelines aiming to support clinical decision-making in the field of rare diseases (RDs). In response to this objective, methodologists and information specialists conducted systematic reviews (SRs). This study aims to explore the barriers/facilitators they encountered.

**Methods:**

A survey was designed to elicit HTA agencies’ experience in developing SRs on RDs. Information was collected on the number of SRs conducted and the types of RDs and clinical questions addressed. In addition, they were asked to identify barriers and facilitators for each stage of the review (from the definition of PICO [population, intervention, comparator, outcome] components of the question to the issuing of recommendations). Finally, they were asked for process improvement suggestions. The survey was distributed by email and completed online. A thematic analysis was conducted to identify the issues identified at each stage of SR.

**Results:**

A total of 111 SRs were conducted on 35 RDs. Most clinical questions were about diagnosis and treatment. The main barriers identified were lack of MesH (Medical Subject Headings) terms associated with the conditions, non-representative abstracts and keywords, lack of relevant information in the body of the articles, and reported data not allowing for quantitative syntheses or recommendations to be made. Facilitating aspects included Orphanet’s specific source of RD documents and having expert clinicians in the working groups who were also involved in all steps of the SR.

**Conclusions:**

Conducting SRs in the field of RDs is challenging. Authors of primary studies are encouraged to be more exhaustive in reporting the results. More research focused on the SR methodology in RDs is necessary to address their particular characteristics and obtain robust results. It is crucial to collaborate with reference networks to address RDs, where the evidence is scarce.

